# Weight-Bearing Ladder Climbing Exercise Improves Bone Loss and Bone Microstructural Damage While Promoting Bone Injury Healing in OVX Rats

**DOI:** 10.3390/biology15010055

**Published:** 2025-12-28

**Authors:** Yiting Kang, Nan Li, Yanan Yu, Dingkang Wang, Tingting Zhao, Lijun Sun, Changjiang Liu, Liang Tang

**Affiliations:** 1 School of Physical Education, Xi’an Jiaotong University, Xi’an 710049, China; kangyiting@xjtu.edu.cn (Y.K.); linan@xjtu.edu.cn (N.L.); 2 Institute of Sports Biology, Shaanxi Normal University, Xi’an 710119, China; limonitenan@foxmail.com (Y.Y.); 15009298730@163.com (T.Z.); sunlijun@snnu.edu.cn (L.S.); 3 Global Health Institute, School of Public Health, Xi’an Jiaotong University, Xi’an 710061, China; kwang@xjtu.edu.cn

**Keywords:** weight-bearing ladder climbing exercise, osteoporosis, ovariectomized, bone injury repair, MSTN/ActRIIB/Smad 3/Wnt/β-catenin pathway

## Abstract

Osteoporosis is highly prevalent in postmenopausal women, causing chronic pain, fractures, and limited mobility that burden individuals and society. While resistance exercise benefits bone health, its role in osteoporotic bone injury healing and underlying mechanisms remain unclear. This study aimed to explore the effects of 10-week weight-bearing ladder climbing exercise on ovariectomy (OVX)-induced osteoporosis and subsequent bone injury healing, and to investigate whether these effects are associated with the myostatin (MSTN) and Wnt/β-catenin pathways. Fifty-four 12-week-old female SD rats were randomized into Sham, OVX, and OVX + EX groups. Rats in the OVX and OVX + EX groups underwent ovariectomy to induce postmenopausal osteoporosis, and those in the OVX + EX group received 10-week weight-bearing ladder climbing. After the exercise intervention, 6 rats in each group were sacrificed; the remaining rats underwent femoral midshaft drilling to establish bone injury. The improvement in osteoporosis was evaluated via Micro-CT, biomechanical tests, RT-qPCR for mRNA detection, and Western blot for measuring protein levels of MSTN and Wnt/β-catenin pathway-related molecules at post-exercise and 21 days post-injury. Bone healing was reflected by the bone volume fraction at the bone injury site detected via Micro-CT at 10 and 21 days post-injury. This exercise significantly enhanced muscle strength and improved femoral bone mineral density (BMD), trabecular microstructure, and biomechanical properties in OVX rats. Meanwhile, the level of MSTN in the OVX + EX group was decreased, the expression of its downstream signaling pathways was inhibited, and the mRNA and protein expressions of Wnt/β-catenin were upregulated. Moreover, 21 days after exercise intervention, the biomechanical properties and bone microstructure of the OVX + EX group were still significantly superior to those of the OVX group, and the aforementioned molecular regulatory effect remained. In addition, pre-conducted exercise was able to promote increases in bone volume fraction at the bone injury site 10 and 21 days after drilling, which was conducive to bone injury healing. Ten-week weight-bearing ladder climbing ameliorates OVX-induced bone loss and promotes osteoporotic bone repair via regulating the MSTN/ActRIIB/Smad3 and Wnt/β-catenin pathways, providing evidence for exercise as a safe non-pharmacological intervention.

## Simple Summary

Postmenopausal women face a high risk of osteoporosis, and the resulting chronic pain, fractures, and limited mobility impose a heavy burden on patients and society. While resistance exercise benefits bone health, it remains unclear whether long-term regular exercise can promote the healing of bone injuries caused by osteoporosis and how it works. This study tested the effects of 10-week weight-bearing ladder climbing in rat models simulating postmenopausal osteoporosis. The results showed that the exercise enhanced muscle strength, balanced hormones related to bone health, and reduced body weight in the osteoporotic rats. It also increased bone density and improved bone structure and strength—importantly, these benefits still existed 21 days after stopping exercise. Furthermore, in bone injury tests conducted after exercise cessation, rats that had undergone prior exercise showed significantly accelerated bone healing. This exercise exerts its effects by lowering a protein that inhibits muscle growth and regulating biological mechanisms that protect bones and muscles, with these effects remaining evident 21 days post-exercise. This study confirms that weight-bearing ladder climbing is a safe, drug-free way to alleviate postmenopausal bone loss, strengthen bones, and aid bone injury healing, providing important support for using exercise as a clinical intervention.

## 1. Introduction

With the gradual intensification of population aging, the incidence of osteoporosis will increase year by year [1,2]. Chronic pain, fractures, and limited mobility caused by osteoporosis impose a huge burden on patients and society [3]. The questions of how to prevent the occurrence of osteoporosis and effectively treat complications after the onset of osteoporosis are urgent issues to be solved by scholars in various fields. Resistance exercise is a major type of exercise that stimulates bone metabolism [4,5]. It can increase stress stimulation on bones, improve the level of bone metabolism, and stimulate bone formation [6]. At present, the research results of many scholars have shown that resistance exercise plays a positive role in the prevention and treatment of osteoporosis [7,8]. However, few studies have explored whether long-term regular resistance exercise can promote the healing of bone injuries after osteoporosis. Furthermore, the mechanism underlying the role of resistance exercise in the prevention and treatment of osteoporosis remains unclear.

Ovariectomy (OVX)-induced bone loss, a well-established animal model of post-menopausal osteoporosis, is characterized by estrogen deficiency-driven disruptions in bone remodeling. Estrogen depletion induces a high bone turnover state, shifting the bone remodeling balance toward increased osteoclast-mediated bone resorption and relatively decreased osteoblast-dependent bone formation. This leads to reduced bone mineral density (BMD), deteriorated trabecular microstructure, and impaired bone biomechanical properties [9,10]. Beyond skeletal deterioration, OVX also triggers systemic metabolic disturbances, including increased body weight gain, skeletal muscle atrophy, and reduced muscle strength, which further exacerbate bone fragility by weakening the “muscle–bone crosstalk”—a bidirectional regulatory axis critical for maintaining skeletal integrity [11,12,13,14]. These pathological changes closely mimic the clinical manifestations of postmenopausal osteoporosis in women, making the OVX rat model a valuable tool for investigating therapeutic strategies to mitigate bone and muscle loss.

Current interventions for postmenopausal osteoporosis primarily include antiresorptive drugs and estrogen replacement therapy, but these approaches are associated with long-term risks such as cardiovascular complications or increased cancer susceptibility [15,16]. In contrast, physical exercise—especially resistance training—has emerged as a safe and cost-effective non-pharmacological strategy to improve bone health. Among various resistance training modalities, resistance ladder climbing exerts combined mechanical stimuli, including axial compression, bending, and shear forces, on weight-bearing bones such as the femur through the synergistic effect of rats’ own body weight and additional loads [17]. This stimulation pattern is highly consistent with the bone loading characteristics during the body’s daily physiological activities, enabling more efficient activation of bone cell mechanotransduction pathways and providing potent physiological stimulation for bone remodeling [17]. Furthermore, the training allows for progressive adjustment of exercise intensity by gradually increasing the load ratio, modifying the number of training sets, and adjusting rest intervals. It not only circumvents the adaptive limitations of single-intensity stimulation but also precisely matches the physiological status of rats to regulate intervention intensity, ensuring the efficacy of the intervention. Therefore, weight-bearing ladder climbing exercise was chosen as the resistance exercise modality in this study. Previous studies have shown that resistance exercise can enhance BMD, preserve trabecular bone volume fraction (BV/TV), and increase bone’s resistance to fracture in OVX animals [18]. However, most existing research focuses on the short-term effects of exercise during or immediately after training, with limited insights into whether these benefits persist after exercise cessation—particularly in the context of bone injury, a common comorbidity in osteoporotic populations. Two key questions therefore remain insufficiently addressed: whether these exercise-induced bone-protective effects persist after cessation and what role prior exercise plays in promoting bone healing in osteoporotic individuals.

The underlying molecular mechanisms linking resistance exercise to sustained bone protection in OVX models remain incompletely understood. Recent evidence highlights the role of myostatin (MSTN), a member of the transforming growth factor-β (TGF-β) superfamily, as a key mediator of muscle–bone crosstalk [18]. MSTN exerts dual inhibitory effects on muscle and bone tissues: it not only suppresses skeletal muscle hypertrophy but also binds to its receptor ActRIIB on osteoblasts and osteoclasts, activating downstream Smad3 signaling to directly inhibit osteoblast differentiation and promote osteoclastogenesis [19,20]. Concurrently, estrogen deficiency in OVX rats downregulates the Wnt/β-catenin pathway—a central regulator of osteogenesis that promotes osteoprogenitor proliferation, inhibits osteoblast apoptosis, and indirectly suppresses osteoclast formation via modulating the OPG/RANKL axis [21]. Resistance exercise has been shown to reduce MSTN expression and activate Wnt/β-catenin signaling in OVX rats [18], but whether this regulatory effect persists post-exercise and contributes to sustained bone protection (including during bone injury repair) in OVX rats has not been systematically investigated.

In the present study, we established a femoral drill-hole injury model in OVX rats—this model is widely recognized for its simplicity, reproducibility, and ability to simulate localized bone defects, making it an ideal tool for evaluating bone repair efficiency in osteoporosis research [22]. We focused on two core objectives: first, to evaluate the ameliorative effects of ten-week weight-bearing ladder climbing exercise on femoral bone mineral density (BMD), trabecular microstructure, and biomechanical properties in OVX rats, to determine whether these effects persist 21 days after the cessation of exercise, and to verify whether the exercise can accelerate the bone repair process following femoral injury in rats; second, to conduct an investigation into the regulatory effects of exercise on the MSTN/ActRIIB/Smad 3 pathway and Wnt/β-catenin signaling pathway, and to reveal their specific mechanisms of action in sustained bone and muscle protection in OVX rats.

## 2. Materials and Methods

### 2.1. Animals

A total of fifty-four 12-week-old female specific pathogen-free (SPF) Sprague-Dawley (SD) rats were obtained from the School of Medicine, Xi’an Jiaotong University (Xi’an, China). These rats were housed 4–5 per cage in individually ventilated cages (IVCs) and maintained in a controlled environment, where they had free access to water and a standard laboratory rodent diet (conforming to China’s national standard GB 14924.3-2010) [23]. The environmental conditions were regulated as follows: temperature was kept at 22 ± 1 °C, relative humidity at 45–50%, and a daily 12 h light/12 h dark cycle (lights on at 08:00, lights off at 20:00) was maintained via automatic control. All experimental procedures were reviewed and approved by the Special Committee on Scientific Ethics of Shaanxi Normal University and strictly adhered to the guidelines outlined in the National Institutes of Health Guide for the Care and Use of Laboratory Animals [24]. All experimental animals were from the same batch and underwent a unified environmental acclimatization period before the experiment to ensure high consistency in their initial physiological status and health level; meanwhile, all experimental treatments and the detection of the same index were performed by the same operator in accordance with a fixed standardized procedure, and the animal cages were randomly placed in the same area of the same rearing room, with the rearing environment kept consistent throughout the experiment.

### 2.2. Animal Grouping and Experimental Design

**Figure 1:**
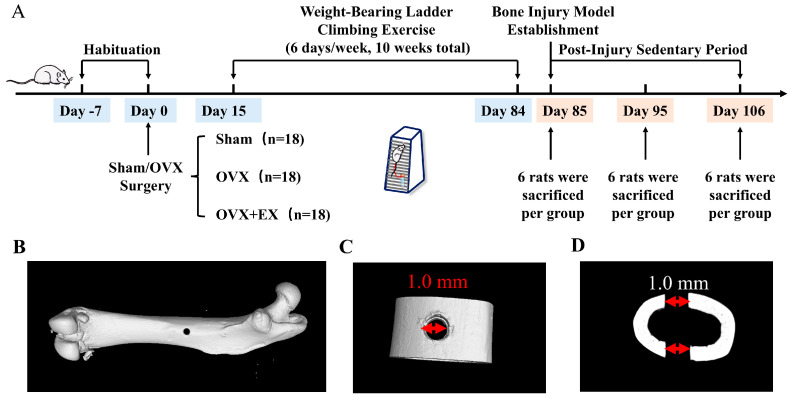
Experimental timeline and Micro-CT reconstruction diagrams of rat injury holes. (**A**) Experimental timeline. (**B**) Overall diagram of the right femur of the rat. (**C**) Three-dimensional diagram of the bone drilling injury site; the red arrows indicate the diameter of the injury hole (1.0 mm). (**D**) Two-dimensional diagram of the bone drilling injury site; the red arrows indicate the diameter of the injury hole (1.0 mm).

The rats were randomly assigned to three experimental groups using a random number table. The Sham-operated group (Sham, *n* = 18): In this group, only adipose tissue of the same volume as the ovaries was excised without removing the ovaries. This group was used to reflect bone metabolic and structural characteristics under normal physiological conditions. The ovariectomized model group (OVX, *n* = 18): The bilateral ovaries of the rats in this group were removed to simulate the bone loss caused by the sudden decline in estrogen levels in postmenopausal women. The ovariectomy + ladder climbing exercise intervention group (OVX + EX, *n* = 18): After bilateral ovariectomy, the rats in this group underwent an additional 10-week weighted ladder—climbing resistance training. This group aimed to evaluate the ameliorative effects of exercise on bone damage, bone loss, and the degradation of bone microstructure induced by ovariectomy. The sample size was determined based on previous experiments, in which the femoral bone mineral density of rats was the primary outcome measure [25]. Two weeks after ovariectomy, when the rats’ wounds had basically healed, the OVX + EX group was subjected to tail-suspended weight-bearing ladder climbing exercise at a frequency of 6 days per week (6 d/w) using a proprietary product independently developed by the laboratory; the exercise ladder was 1 m high with an inclination angle of 85° and a 2 cm interval between each rung. During the first 2 weeks of exercise, the rats followed a regimen of 3 sets per day (3 sets/d) with 5 repetitions per set (5 reps/set) and a 2 min rest between sets, with the weight load set at 35% of their own body weight; from the 3rd to the 6th week of training, the weight load was increased to 50% of their body weight, and the regimen was adjusted to 3 sets/d with 7 reps/set (still 2 min of rest between sets); from the 7th to the 10th week, the weight load was further raised to 70% of their body weight, and the regimen was 3 sets/d with 10 reps/set (2 min rest between sets remained unchanged). Rats in the other groups received no exercise intervention. Twenty-four hours after the last exercise, 6 rats in each group were sacrificed, and their serum and tissues were collected for subsequent detection; for the remaining rats, a bone injury model was established on the right femur, and on days 10 and 21 post-bone injury modeling, 6 rats in each group were sacrificed, with serum and tissues collected for subsequent detection. Blinding was not implemented in this experiment. The experimental timeline is shown in Figure 1A.

### 2.3. Ovariectomy

Rats were anesthetized via intraperitoneal injection of 3% pentobarbital sodium at 50 mg/kg. For rats assigned to the OVX group, ovariectomy was performed as follows: bilateral incisions were created through the skin and musculature on the dorsolateral lumbar region, slightly ventral to the lumbar vertebrae. After opening the peritoneum, the ovaries along with their associated adipose tissue were exteriorized; subsequently, the uterine horns and ovarian blood vessels were ligated, and the ovaries were excised. The incisions were then closed using absorbable sutures. As for the Sham group, rats underwent a sham ovariectomy procedure, in which only a portion of the adipose tissue surrounding the ovaries was removed, while the ovaries themselves were retained in their original anatomical position. In the postsurgical period, the incision sites were disinfected daily with Anerdian skin disinfectant for a consecutive 3-day period.

### 2.4. Establishment of the Bone Injury Model

The bone injury model was established according to the protocol described by He et al [22]. All instruments were sterilized by autoclaving or alcohol to ensure aseptic operation. Anesthesia was induced via intraperitoneal injection of 3% pentobarbital sodium at a dose of 50 mg/kg. After adequate anesthesia, the hair on the right hindlimb was shaved to expose the skin, and the rat was fixed in a lateral position on the operating table. The skin was pre-treated with 75% ethanol. A longitudinal incision was made on the lateral side of the right hindlimb, and the skin, subcutaneous tissue, and muscles were bluntly dissected along the direction of muscle fibers to fully expose the femur. A 1.0 mm drill bit was used to penetrate the bone at the midshaft of the right femur. After drilling, the femur and wound were rinsed with sterile normal saline to remove bone debris, and it was confirmed that both cortical bones were penetrated. The incision was sutured layer by layer using sterile surgical needles and sutures pre-soaked in normal saline. To prevent postoperative infection, the wound was wiped with iodophor-soaked cotton balls. For analgesia, buprenorphine (0.1 mg/kg) was administered intraperitoneally immediately after surgery and repeated 24 h later to ensure no significant pain stress in the animals. Postoperatively, the animals’ mental status, activity, food/water intake, and surgical site (for redness, swelling, or infection) were monitored daily for 7 consecutive days. No obvious pain responses or infections were observed in any animals during the experiment. Notably, the injury model did not require additional fixation, and rats regained full mobility within 1–2 days postoperatively. The injury model is illustrated in Figure 1B–D. All drilling procedures were performed by the same experimenter following standardized protocols to ensure consistent injury severity across groups.

### 2.5. Sample Collection

After completing the experimental treatment, the rats were euthanized via an overdose injection of pentobarbital. Blood was collected using the abdominal aorta puncture method; following a 90 min incubation at room temperature, the blood samples were centrifuged at 1500× *g* for 20 min at 4 °C. The serum was then separated and stored at −80 °C for subsequent analysis. The left quadriceps femoris of each rat was isolated, weighed, then placed in liquid nitrogen overnight before being transferred to a −80 °C environment for storage. The left and right femurs of the rats were isolated, and the adhering soft tissues were removed: the right femur was wrapped in gauze soaked with normal saline, placed on ice, and then transferred to a −20 °C environment for storage, which was used for micro-computed tomography (micro-CT) and biomechanical analysis; the left femur was placed in liquid nitrogen overnight, then transferred to −80 °C for storage and used for quantitative real-time PCR analysis. The uterus of each rat was isolated and weighed immediately. No animals or data were excluded in this experiment.

### 2.6. Serum Analysis

Enzyme-Linked Immunosorbent Assay (ELISA) kits (Cusabio, Wuhan, China; Cayman, Ann Arbor, MI, USA) were used to determine the serum levels of myostatin (MSTN) and estradiol (E_2_). Serum alkaline phosphatase (ALP) and tartrate-resistant acid phosphatase (TRACP) concentrations were analyzed via a standard colorimetric method using commercial kits (Nanjing Jiancheng Bioengineering Institute, Nanjing, China), following the protocols supplied by the manufacturers. Absorbance measurements were performed using a Model 680 microplate reader (Bio-Rad Corporation, Philadelphia, PA, USA).

### 2.7. Grip Strength

Grip strength of the rats was measured using a grip strength meter (Model YLS-13A, Anhui Zhenghua Bioinstrumentation Co., Ltd.; Huaibei, Anhui, China). Each rat underwent three consecutive tests with no rest intervals between trials, and the results of these three measurements were averaged to obtain the final grip strength value for each individual rat.

### 2.8. Dual-Energy X-Ray Absorption (DXA) and Micro-Computed Tomography (Micro-CT)

In vivo bone mineral density (BMD) of the bilateral femurs in rats was measured using a dual-energy X-ray system (MEDIKORS InAlyzer, Seongnam-si, Republic of Korea). Micro-computed tomography (micro-CT; Y. Cheetah; YXLON International GmbH, Hamburg, Germany) was employed to assess the effect of exercise on microarchitectural deterioration associated with OVX-induced bone loss. Samples, wrapped in gauze soaked with normal saline to maintain moisture, were thawed at room temperature. For femur scanning and imaging, VG Studio 2.2 V software (Version 2.2) was used with an X-ray tube set to 80 kV (voltage) and 60 μA (current), and a pixel size of 18 μm. A total of 450 projection images were collected over a 180° angular range, after which image slices were reconstructed via cone-beam reconstruction software (SkyScan, Bruker microCT N.V, Kontich, Belgium) based on the Feldkamp algorithm. The region of interest (ROI) was defined as a 2 mm segment starting 0.3 mm proximal to the end of the growth plate. Trabecular bone was isolated by drawing custom contours using CT analyzer software (VG Studio MAX 2.2, Volume Graphics GmbH, Heidelberg, Germany). From the 3D-rendered micro-CT images, key parameters were extracted: BMD, trabecular thickness (Tb.Th), trabecular bone volume fraction (BV/TV), trabecular number (Tb.N), and trabecular separation (Tb.Sp). All 3D parameters were derived from the analysis of a marching cubes-type model with a rendered surface.

### 2.9. Biomechanical Testing

Three-point bending tests were performed to evaluate both extrinsic and intrinsic biomechanical properties of the samples. Samples were wrapped in gauze soaked with normal saline (to maintain moisture) and thawed at room temperature. The thawed right femurs were subjected to mechanical testing until failure using a material testing system (MTS-858, MTS Systems Corporation, Minneapolis, MN, USA). The load was applied to the midshaft of the femur, with a 10 mm distance between the two supports (measured from the midpoints of the supports). Load–displacement curves and real-time force changes were recorded at a constant crosshead speed of 2 mm/min until femoral fracture. Geometric parameters at the fracture site of each femur were measured using a vernier caliper: aouter (outer major axis), bouter (outer minor axis), ainner (inner major axis), and binner (inner minor axis). Key biomechanical parameters, including ultimate load, stiffness (linear slope), and energy absorption, were directly derived from the load-deformation curves. Elastic modulus was calculated using the formula proposed by Turner & Burr [26]: E = FL^3^/48 dI where E is the elastic modulus (MPa), F is the maximum load (N), L is the distance between the two supports (mm), d is the displacement (mm), and I is the moment of inertia of the femoral cross-section relative to the horizontal axis (mm^4^). Cortical bone cross-sectional area (CB CSA) was computed using the elliptical ring model: CB CSA = π/4(a_outer_b_outer_ − a_inner_b_inner_). To eliminate confounding effects of bone structural size on biomechanical parameters (and to reflect material properties per unit area of cortical bone), the ultimate load, stiffness, and energy absorption were normalized as follows: Normalized ultimate load = Ultimate load (N)/CB CSA (mm^2^), Normalized stiffness = Stiffness (N/mm)/CB CSA (mm^2^), Normalized energy absorption = Energy absorption (mJ)/CB CSA (mm^2^).

### 2.10. RNA Isolation and Real-Time Polymerase Chain Reaction

Total RNA was extracted from femoral tissues using the EASYspin Plus Bone Tissue RNA Kit (Aidlab, Beijing, China). Gene expression analysis was conducted with the CFX96 Real-Time PCR Detection System (BIO-RAD Laboratories, Hercules, CA, USA). Reverse transcription was carried out using the PrimeScript™ RT Reagent Kit with gDNA Eraser (Takara, Kusatsu, Japan), while RT-qPCR quantification of mRNA expression and remodeling markers was performed with the TB Green^®^ Premix Ex Taq™ II (Takara, Kusatsu, Japan). The primers utilized are detailed in Table 1. Amplification reactions consisted of 45 cycles with the following parameters: 95 °C for 10 min, 95 °C for 15 s, 60 °C for 1 min, and 72 °C for 30 s. Glyceraldehyde-3-phosphate dehydrogenase (*GAPDH*) served as the housekeeping gene for normalization purposes. Relative changes in gene expression were analyzed using the 2^(−ΔΔCt)^ method. The calculation steps are as follows. First, ΔCt for each sample (Ct of target gene − Ct of reference gene) is calculated using the mean of technical replicates. Second, the Sham group is used as the calibrator to calculate ΔΔCt (ΔCt of experimental group − mean ΔCt of Sham group). Third, 2^(−ΔΔCt)^ is derived as the relative expression level. All experiments were performed with 3 biological replicates, and each biological sample was simultaneously subjected to 3 technical replicates.

### 2.11. Western Blot Analysis

The left femurs were homogenized in ice-cold lysis buffer. Protein concentrations were quantified using the BCA Protein Assay Kit (Thermo Scientific, Waltham, MA, USA). Equal amounts of total protein were separated by SDS-polyacrylamide gel electrophoresis (SDS-PAGE) on 8–12% polyacrylamide gels and then transferred to nitrocellulose membranes. The immunoblots were incubated with primary antibodies overnight at 4 °C, followed by incubation with corresponding secondary antibodies at room temperature for 2 h. Blot visualization was performed using ECL-plus chemiluminescent reagent, and the results were quantitatively analyzed with Lab Image version 2.7.1 software. The primary antibodies used were as follows: Myostatin (MSTN, Cat. No. bs-1288R), Activin Type II B Receptor (ActRIIB, Cat. No. bs-12417R), and Glycogen Synthase Kinase 3β (GSK-3β, Cat. No. bsm-63070R) purchased from Bioss Inc. (Beijing, China); Wnt1 (Cat. No. 27935–1-AP) and β-Catenin (Cat. No. 66379–1-Ig) purchased from Proteintech Group Inc. (Wuhan, Hubei, China); and Smad3 (Cat. No. ET1607-41) purchased from HUABIO Inc (Hangzhou, Zhejiang, China).

### 2.12. Statistical Analysis

The experimental results are presented as mean ± standard deviation (mean ± SD). G*Power 3.1 software (Franz Faul, Düsseldorf, Germany) was applied for statistical power analysis of the primary outcome measure, femoral bone mineral density (BMD). With the significance level set at α = 0.05, number of groups at k = 3, and sample size per group at *n* = 6, the calculated Cohen’s f (effect size) was 1.08. According to Cohen’s criteria, this value represents an extremely large effect size (criteria: f ≥ 0.4 indicates a large effect size), yielding an achieved statistical power (1 − β) of 0.99. The Kolmogorov–Smirnov test was used to verify the normality of the data distribution. If the data conformed to a normal distribution, one-way analysis of variance (one-way ANOVA) was applied, followed by Tukey’s post hoc test for pairwise comparisons between groups; if the data did not conform to a normal distribution, the Kruskal–Wallis test was used instead. The statistical significance levels were set at *p* < 0.05, *p* < 0.01, and *p* < 0.001. All statistical analyses were performed using SPSS 25.0 s (IBM, Armonk, NY, USA) and GraphPad Prism 9.0 (GraphPad, Inc., La Jolla, CA, USA).

## 3. Results

### 3.1. Weight-Bearing Ladder Climbing Improves Serum Hormone Levels, Bone Metabolism Indicators, Tissue Weights and Grip Strength in OVX Rats

**Figure 2:**
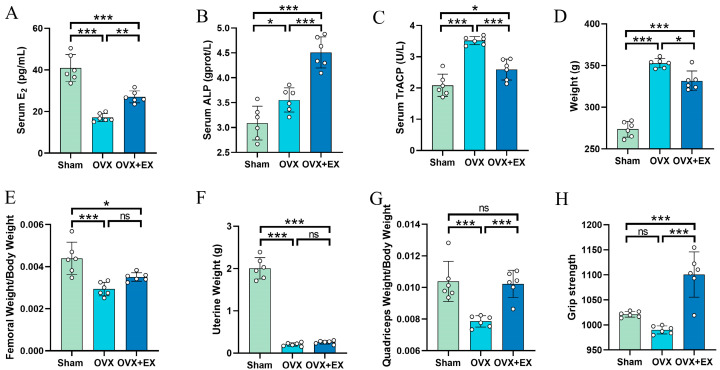
Effects of weight-bearing ladder climbing exercise intervention on serum hormones, bone metabolism indicators, related tissue weights and grip strength in OVX rats. (**A**) Serum E_2_ (estradiol). (**B**) Serum ALP (alkaline phosphatase). (**C**) Serum TrACP (serum tartrate-resistant acid phosphatase). (**D**) Body weight. (**E**) Femoral Weight/Body Weight. (**F**) Uterine Weight. (**G**) Quadriceps Weight/Body Weight. (**H**) Grip strength. Statistical analysis was performed using one-way ANOVA followed by Tukey’s post hoc test for pairwise comparisons; *n* = 6 biologically independent samples; *** *p* < 0.001, ** *p* < 0.01, * *p* < 0.05; ns means no significance. Data are represented as means ± SD.

As shown in Figure 2A, serum E_2_ levels were significantly lower in the OVX group compared to the Sham group, whereas the OVX + EX group exhibited a significant increase relative to the OVX group. Similarly, Figure 2B,C demonstrate that serum alkaline phosphatase (ALP) and tartrate-resistant acid phosphatase 5b (TRACP) levels were significantly higher in the OVX group than in the Sham group. Compared with the OVX group, the OVX + EX group showed a significant increase in ALP levels alongside a significant decrease in TRACP levels. Figure 2D reveals that the OVX group had significantly higher body weight than the Sham group, while the OVX + EX group displayed reduced body weight compared to the OVX group. In terms of bone-related parameters, Figure 2E shows that the femur weight/body weight ratio was significantly lower in the OVX group than in the Sham group; however, the OVX + EX group only showed a non-significant increasing trend. Figure 2F indicates that uterine weight was significantly lower in the OVX group compared to the Sham group, with no significant difference observed between the OVX + EX and OVX groups. Regarding muscle mass, Figure 2G illustrates that the quadriceps femoris weight/body weight ratio was significantly lower in the OVX group than in the Sham group, but this ratio increased significantly in the OVX + EX group compared to the OVX group. Finally, Figure 2H shows that while grip strength tended to decrease in the OVX group relative to the Sham group, this difference was not statistically significant. In contrast, the OVX + EX group exhibited significantly higher grip strength than the OVX group.

### 3.2. Weight-Bearing Ladder Climbing Enhances Femoral BMD and Optimizes Trabecular Microstructure in OVX Rats

**Figure 3:**
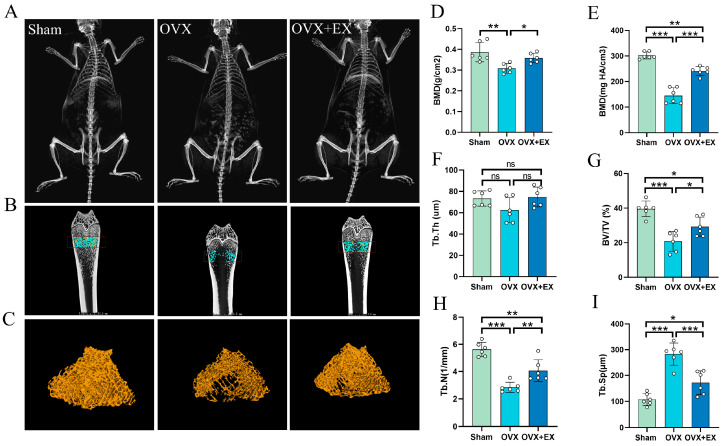
Effects of weight-bearing ladder climbing exercise intervention on femoral BMD and bone microstructure in rats. (**A**) Representative whole-body X-ray images of rats. (**B**) Representative images of distal femur in each group. (**C**) Three-dimensional reconstruction images of trabecular bone in distal femur of each group. (**D**) The BMD of whole femur. (**E**) The BMD of distal femur. (**F**) The trabecular number (Tb.N) of distal femur. (**G**) The bone volume/tissue volume (BV/TV) of distal femur. (**H**) The trabecular thickness (Tb.Th) of distal femur. (**I**) The trabecular separation (Tb.Sp) of distal femur. Statistical analysis was performed using one-way ANOVA followed by Tukey’s post hoc test for pairwise comparisons; *n* = 6 biologically independent samples; *** *p* < 0.001, ** *p* < 0.01, * *p* < 0.05; ns means no significance. Data are represented as means ± SD.

To investigate the effect of exercise intervention on BMD and bone microstructure in OVX rats, this study measured the femoral BMD and distal femoral bone microstructure of the rats. The results showed that compared with the OVX group, the femoral BMD and bone microstructure were improved in the resistance ladder climbing OVX + EX group (Figure 3A–I). Specifically, compared with the Sham group, the total femoral BMD and distal femoral cancellous BMD in the OVX group were significantly decreased, whereas the above two BMD indicators in the OVX + EX group were significantly higher than those in the OVX group. Regarding bone microstructure parameters, compared with the Sham group, the trabecular number (Tb.N) and bone volume fraction (BV/TV) in the OVX group were significantly reduced, while these two parameters in the OVX + EX group were significantly higher than those in the OVX group. No significant difference in trabecular thickness (Tb.Th) was detected among the three groups. In addition, resistance ladder climbing training significantly inhibited the increase in trabecular separation (Tb.Sp) in the OVX group.

### 3.3. Weight-Bearing Ladder Climbing Improves Femoral Biomechanical Properties in OVX Rats

**Figure 4:**
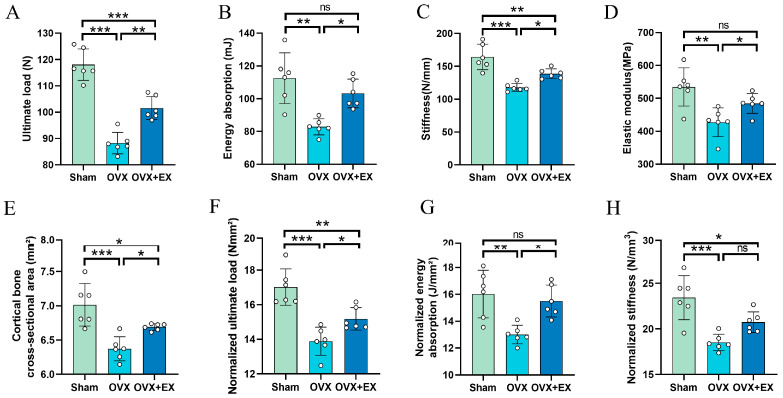
Effects of weight-bearing ladder climbing exercise intervention on femoral biomechanical properties in OVX rats. (**A**) Ultimate load. (**B**) Energy absorption. (**C**) Stiffness. (**D**) Elastic modulus. (**E**) Cortical bone cross-sectional area. (**F**) Normalized ultimate load. (**G**) Normalized energy absorption. (**H**) Normalized stiffness. Statistical analysis was performed using one-way ANOVA followed by Tukey’s post hoc test for pairwise comparisons; *n* = 6 biologically independent samples; *** *p* < 0.001, ** *p* < 0.01, * *p* < 0.05; ns means no significance. Data are represented as means ± SD.

To investigate the effect of exercise on femoral biomechanical properties in OVX rats, we measured ultimate load, energy absorption, stiffness, elastic modulus, and cortical bone cross-sectional area (CB CSA), and normalized the first three parameters by CB CSA. As shown in Figure 4, compared with the Sham group, the OVX group exhibited significant reductions in ultimate load, energy absorption, stiffness, elastic modulus and CB CSA. In contrast, 10-week weight-bearing ladder climbing exercise reversed these deficits; compared with the OVX group, the OVX + EX group showed improved ultimate load, energy absorption, stiffness, elastic modulus and CB CSA. Normalization analysis revealed that the OVX group still had significantly lower normalized ultimate load, normalized stiffness, and normalized energy absorption than the Sham group. In the OVX + EX group, normalized ultimate load and energy absorption were significantly higher than in the OVX group, but normalized stiffness showed no difference.

### 3.4. Weight-Bearing Ladder Climbing Regulates MSTN Expression and Activates Femur-Related Signaling Pathways in OVX Rats

**Figure 5:**
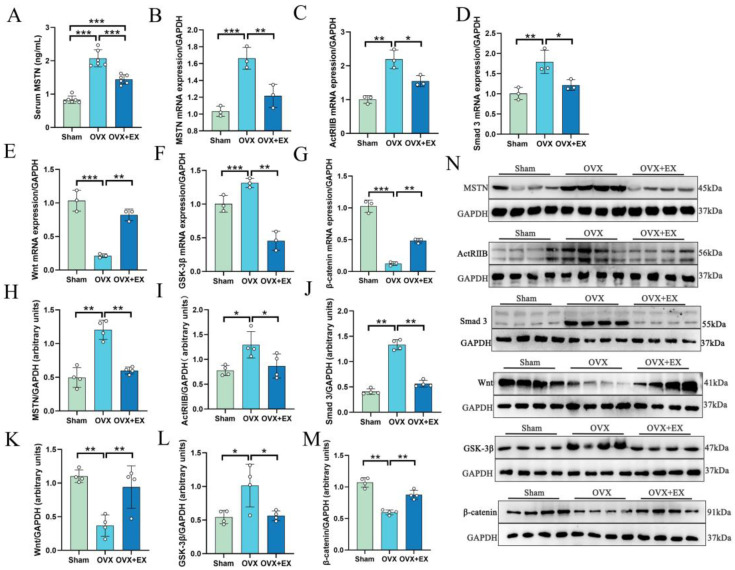
Effects of weight-bearing ladder climbing exercise intervention on serum MSTN level, MSTN mRNA expression in quadriceps femoris, and mRNA and protein expression of MSTN/ActRIIB/Smad3/Wnt/β-catenin pathway-related molecules in femur of OVX rats. (**A**) Serum MSTN. (**B**) *MSTN* mRNA expression. (**C**) *ActRIIB* mRNA expression. (**D**) *Smad* 3 mRNA expression. (**E**) *Wnt* mRNA expression. (**F**) *GSK-3β* mRNA expression. (**G**) *β-catenin* mRNA expression. (**H**) Related protein expression of MSTN. (**I**) Related protein expression of ActRIIB. (**J**) Related protein expression of Smad 3. (**K**) Related protein expression of Wnt. (**L**) Related protein expression of GSK-3β. (**M**) Related protein expression of β-catenin. (**N**) Western blot images. Statistical analysis was performed using one-way ANOVA followed by Tukey’s post hoc test for pairwise comparisons; *n* = 3 or 4 biologically independent samples; *** *p* < 0.001, ** *p* < 0.01, * *p* < 0.05. Data are represented as means ± SD. The original Western blot images were summarized in Table S1.

To investigate the molecular mechanism by which exercise improves BMD and bone microstructure in OVX rats, we examined the serum MSTN level, MSTN protein expression in the quadriceps femoris, and the mRNA and protein expression of femoral-related signaling pathway molecules in OVX rats. As shown in Figure 5, compared with the Sham group, the serum MSTN level and the relative mRNA and protein expression of MSTN in the quadriceps femoris of OVX rats were significantly increased; 10-week resistance ladder climbing exercise significantly inhibited the expression of the aforementioned indicators. Further detection of the femoral MSTN receptor ActRIIB showed that its mRNA and protein expression were significantly upregulated in OVX rats but remarkably inhibited in OVX + EX rats. The expressions of its downstream molecules *Smad3* and *GSK-3β* mRNA and protein were also significantly higher in the OVX group than in the Sham group, and significantly lower in the OVX + EX group than in the OVX group. The expressions of Wnt and β-catenin mRNA and protein were significantly lower in the OVX group than in the Sham group, and significantly higher in the OVX + EX group than in the OVX group.

### 3.5. Prior Weight-Bearing Ladder Climbing Promotes Bone Injury Healing in OVX Rats

**Figure 6:**
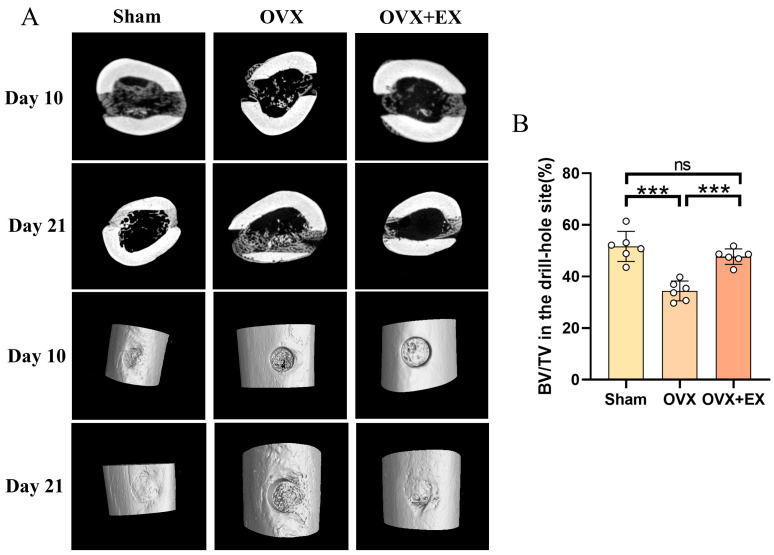
Effects of weight-bearing ladder climbing exercise intervention on bone injury healing in OVX rats. (**A**) Representative Micro-CT images of drill-hole sites in femurs from Sham, OVX, and OVX + EX groups at Day 10 and Day 21. (**B**) Bone volume/tissue volume (BV/TV) at drill-hole sites. Statistical analysis was performed using one-way ANOVA followed by Tukey’s post hoc test for pairwise comparisons; *n* = 6 biologically independent samples; *** *p* < 0.001; ns means no significance. Data are represented as means ± SD.

To investigate the effect of early exercise intervention on bone injury healing in OVX rats, we performed Micro-CT scans on the 10th and 21st days after femoral injury to evaluate bone repair at the drilling site. As shown in Figure 6A, on the 10th post-operative day (Day 10), the Sham group exhibited relatively favorable bone tissue repair at the drilling site with sufficient bone filling; the OVX group showed obvious insufficient bone filling at the drilling site accompanied by numerous porelike structures, while the OVX + EX group had improved bone filling at the drilling site compared with the OVX group, which was more similar to the Sham group. On the 21st post-operative day (Day 21), the Sham group showed further repair of bone tissue at the drilling site with a more complete structure; the OVX group still had unsatisfactory bone tissue repair at the drilling site with sparse bone tissue; and the OVX + EX group displayed significantly enhanced bone tissue repair at the drilling site and increased bone tissue density compared with the OVX group. Quantitative analysis results at 21 days after drilling (Figure 6B) showed that compared with the Sham group, the bone volume fraction at the injury site of OVX rats was significantly decreased, indicating reduced bone formation capacity at the drilling site after ovariectomy. In contrast, the bone volume fraction in the OVX + EX group was significantly higher than that in the OVX group, suggesting that early exercise intervention can effectively mitigate the ovariectomy-induced decrease in bone volume fraction at the drilling site and promote bone repair.

### 3.6. Prior Weight-Bearing Ladder Climbing Still Exerts Significant Improvements on Femoral Cancellous BMD and Microstructure in OVX Rats at 21 Days Post-Exercise Cessation

**Figure 7:**
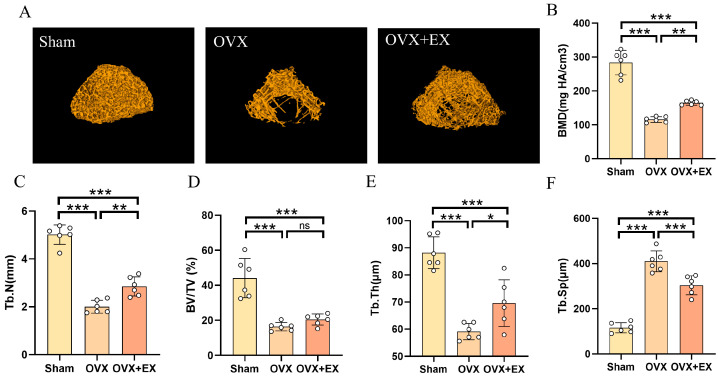
Effect of early exercise intervention on distal femoral microstructure and bone mineral density in ovx rats at 21 days after bone injury. (**A**) Representative three-dimensional reconstruction images of trabecular bone on the distal femur. (**B**) Bone mineral density (BMD). (**C**) The trabecular number (Tb.N) of distal femur. (**D**) The bone volume/tissue volume (BV/TV) of distal femur. (**E**) The trabecular thickness (Tb.Th) of distal femur. (**F**) The trabecular separation (Tb.Sp) of distal femur. Statistical analysis was performed using one-way ANOVA followed by Tukey’s post hoc test for pairwise comparisons; *n* = 6 biologically independent samples; *** *p* < 0.001, ** *p* < 0.01, * *p* < 0.05; ns means no significance. Data are represented as means ± SD.

Twenty-one days after bone injury, we re-examined changes in the microstructure of the distal femur in rats, with results shown in Figure 7A. Twenty-one days after bone injury and cessation of exercise, the BMD and bone microstructure of the distal femur in the OVX group further deteriorated: the 3D structure of bone tissue was significantly sparse, with massive trabecular bone fractures, increased pores, and severe destruction of structural integrity. In contrast, the 3D structure of bone tissue in the OVX + EX group was significantly improved compared with the OVX group, and the density and connectivity of trabecular bone were more similar to those in the Sham group, suggesting that exercise intervention can effectively enhance the integrity of bone microstructure. Quantitative analysis results showed that in the cancellous bone region of the OVX group, BMD, Tb.N, BV/TV and Tb.Th were significantly decreased, while Tb.Sp was significantly increased (Figure 7B–F). However, in rats that received exercise intervention before injury, the deterioration of bone microstructure was significantly inhibited. Specifically, compared with the OVX group, these rats had a higher Tb.N, greater Tb.Th, and smaller Tb.Sp in the cancellous bone region, while no significant change was observed in BV/TV.

### 3.7. Prior Weight-Bearing Ladder Climbing Still Significantly Improves the Distal Femoral Microstructure in OVX Rats 21 Days After Exercise Cessation

**Figure 8:**
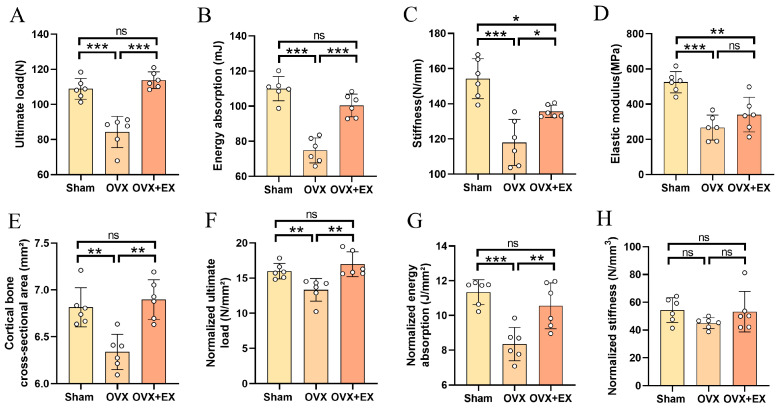
Effects of early exercise intervention on femoral biomechanical properties in OVX rats. (**A**) Ultimate load. (**B**) Energy absorption. (**C**) Stiffness. (**D**) Elastic modulus. (**E**) Cortical bone cross-sectional area. (**F**) Normalized ultimate load. (**G**) Normalized energy absorption. (**H**) Normalized stiffness. Statistical analysis was performed using one-way ANOVA followed by Tukey’s post hoc test for pairwise comparisons; *n* = 6 biologically independent samples; *** *p* < 0.001, ** *p* < 0.01, * *p* < 0.05; ns means no significance. Data are represented as means ± SD.

Similarly, 21 days after bone injury, we reevaluated the changes in femoral mechanical properties of rats, with results presented in Figure 8. The protective effect of early exercise on femoral mechanical properties in rats persisted, which was specifically reflected in significantly higher ultimate load, energy absorption, stiffness, cortical bone cross-sectional area, normalized ultimate load, and normalized energy absorption in the OVX + EX group compared with the OVX group. Notably, the exercise-induced improvement in elastic modulus was not sustained, with no significant difference between the OVX + EX and OVX groups. Similarly, normalized stiffness showed no significant difference between these two groups.

### 3.8. The Regulatory Effects of Prior Weight-Bearing Ladder Climbing on MSTN and Femur-Related Signaling Pathways Remain Significant 21 Days After Exercise Cessation

**Figure 9:**
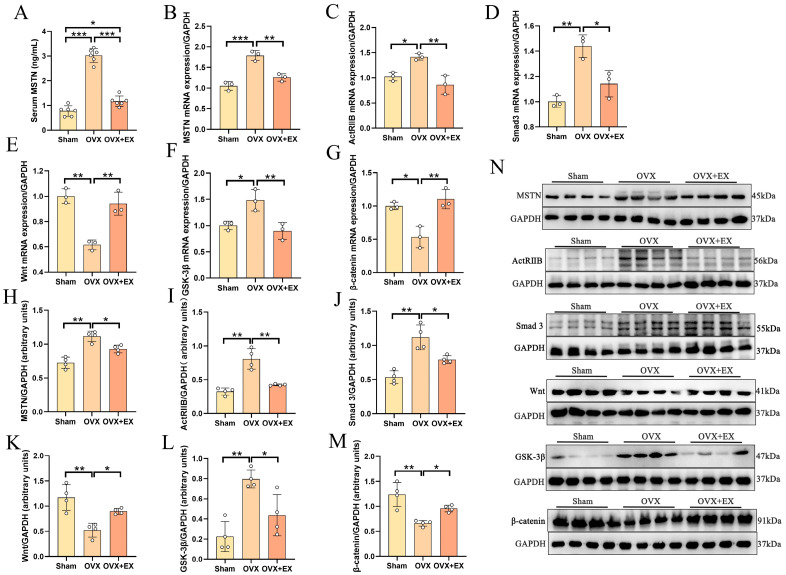
Effects of early exercise intervention on serum MSTN, quadriceps femoris *MSTN* and femur-related signaling pathway gene and protein expression in OVX rats. (**A**) Serum *MSTN*. (**B**) *MSTN* mRNA expression. (**C**) *ActRIIB* mRNA expression. (**D**) *Smad* 3 mRNA expression. (**E**) *Wnt* mRNA expression. (**F**) *GSK-3β* mRNA expression. (**G**) *β-catenin* mRNA expression. (**H**) Related protein expression of MSTN. (**I**) Related protein expression of ActRIIB. (**J**) Related protein expression of Smad 3. (**K**) Related protein expression of Wnt. (**L**) Related protein expression of GSK-3β. (**M**) Related protein expression of β-catenin. (**N**) Western blot images. Statistical analysis was performed using one-way ANOVA followed by Tukey’s post hoc test for pairwise comparisons; *n* = 3 or 4 biologically independent samples; *** *p* < 0.001, ** *p* < 0.01, * *p* < 0.05. Data are represented as means ± SD. The original Western blot images were summarized in Table S2.

As shown in Figure 9, twenty-one days after ceasing exercise, we re-detected the circulating MSTN level, *MSTN* mRNA and protein expression in the quadriceps femoris, and MSTN-related signaling pathway molecules in the femur.

The results are shown in Figure 9: the serum MSTN content and *MSTN* mRNA and protein expression in the quadriceps femoris of rats in the OVX + EX group were still significantly lower than those in the OVX group; the mRNA and protein expression of its receptor ActRIIB and downstream molecules Smad3 and GSK-3β were still significantly inhibited, while the mRNA and protein expression of Wnt and β-catenin were still significantly upregulated.

## 4. Discussion

This study systematically investigated the effects of ten weeks weight-bearing ladder climbing exercise on bone metabolism, muscle strength, and bone injury repair in OVX rats, and explored the underlying molecular mechanisms. The key findings are as follows: weight-bearing ladder climbing exercise ameliorates OVX-induced estrogen deficiency, regulates bone metabolism markers, reduces abnormal weight gain, and mitigates skeletal muscle atrophy. Additionally, this exercise improves femoral BMD, preserves trabecular microstructure, and enhances bone biomechanical properties, with these protective effects still detectable at least 21 days after exercise cessation. Furthermore, it accelerates bone repair after femoral injury. Mechanistically, the sustained benefits of this exercise are likely to be associated with sustained inhibition of the MSTN/ActRIIB/Smad3 catabolic pathway and activation of the Wnt/β-catenin anabolic pathway. Collectively, these results highlight the potential of weight-bearing ladder climbing exercise as a non-pharmacological strategy for postmenopausal osteoporosis and osteoporotic bone injury.

Ovariectomy-induced estrogen deficiency disrupts systemic homeostasis, which is characterized by reduced serum E_2_, elevated bone turnover markers, abnormal weight gain, and skeletal muscle atrophy, and these changes further weaken muscle–bone crosstalk and exacerbate bone fragility [27]. In this study, we observed that 10 weeks of weight-bearing ladder climbing exercise significantly increased serum E_2_ levels in OVX rats, which aligns with previous findings that exercise can upregulate aromatase activity in adipose tissue and skeletal muscle, promoting local estrogen synthesis [28,29]. Notably, exercise also reversed OVX-induced muscle atrophy: the quadriceps femoris weight/body weight ratio and grip strength were significantly higher in the OVX + EX group than in the OVX group. This is consistent with the role of resistance exercise in stimulating muscle protein synthesis via mechanical stress [30]. Importantly, improved muscle mass and strength may indirectly protect bone by enhancing mechanical loading on the skeleton, which serves as an essential stimulus for osteoblast proliferation and bone formation [31,32].

BMD and trabecular microstructure are key determinants of bone strength, while biomechanical properties directly reflect bone’s ability to resist fracture [33,34]. Our results showed that OVX rats exhibited significantly reduced total femoral BMD, distal femoral cancellous BMD, Tb.N, and BV/TV, along with increased Tb.Sp—consistent with the pathological features of postmenopausal osteoporosis [35]. Exercise reversed these changes, which is in line with previous studies showing that resistance exerciseenhances bone formation by increasing mechanical stress on bone tissue [36,37]. Although trabecular thickness (Tb.Th) did not reach statistical significance in this study, the mean Tb.Th in the OVX + EX group showed a tendency to increase compared with the OVX group (74.75 ± 9.27 vs. 62.51 ± 11.72 μm). Combined with the significant improvements in other parameters, these findings collectively support the protective effect of resistance ladder climbing exercise on the trabecular microstructure in ovariectomized rats. The results of biomechanical testing further revealed that the improvement of bone biomechanical properties by resistance exercise is characterized by the synergistic effect of structural enhancement and material optimization. On one hand, the increase in CB CSA serves as a typical structural adaptation [38], laying an anatomical foundation for the improvement of overall bone bearing capacity. On the other hand, the normalized ultimate load and energy absorption (normalized by CB CSA) remained superior, which provides direct evidence that exercise simultaneously optimizes the strength and toughness of bone material per unit area. However, the absence of significant differences in normalized stiffness between groups suggests that the intrinsic deformation resistance of bone material was not significantly altered by exercise, and the improvement in overall stiffness mainly relied on the thickening and strengthening of cortical bone structure. This improvement mode, dominated by structural adaptations and coordinated by material improvements, not only reflects the positive regulation of exercise on bone mass accumulation and morphological remodeling but also reveals the differential response mechanisms of structural and material properties in bone biomechanics to exercise stimulation.

An important finding of this study is that the beneficial effects of exercise were still evident at 21 days after cessation. Even after stopping exercise and subsequent bone injury, compared with the OVX group, the OVX + EX group still maintained higher BMD, superior trabecular microstructure—characterized by increased Tb.Th and Tb.N as well as decreased Tb.Sp—but no significant change in BV/TV. This reflects the temporal heterogeneity and structural specificity of exercise-induced bone adaptation. As local structural parameters, the improvements in Tb.Th and other indices might be sustained by epigenetically mediated cellular memory, with significant differences still observed at 3 weeks post-exercise [39,40]; however, such improvements were only partial and limited in magnitude. In contrast, as a global quantitative parameter, BV/TV relies on continuous bone matrix deposition. Since mechanical stimulation diminishes after exercise cessation, and osteogenic activity as well as bone matrix deposition rate decrease over time, these partial local improvements may fail to translate into a significant increase in BV/TV [39,41].

Similarly, superior bone biomechanical properties were still retained, as evidenced by higher values of ultimate load, energy absorption, and stiffness. Notably, the high levels of normalized ultimate load and normalized energy absorption were also sustained. This persistent effect addresses a critical limitation of existing research—most current studies only focus on the immediate effects after exercise intervention, with insufficient exploration of the sustainability of beneficial effects following exercise cessation. The sustained improvement in bone biomechanical properties, especially bearing capacity and energy absorption, indicates that exercise may induce adaptive remodeling of bone tissue. It is worth noting that the improvement in elastic modulus induced by exercise was not sustained, and normalized stiffness showed no significant difference between the two groups throughout the study. As an indicator reflecting the intrinsic elastic properties of bone material, elastic modulus is mainly determined by the mineral-to-collagen ratio [42]. The transient nature of this effect suggests that exercise-induced changes in bone material composition may require continuous mechanical stimulation to be maintained, whereas structural adaptations exhibit higher stability. This selective persistence implies that although exercise can provide short-term sustained effects structural protection for bone tissue, regular maintenance training may be necessary to preserve the improvements in material-level properties. This finding provides important insights for formulating exercise intervention strategies for postmenopausal osteoporosis, emphasizing the need to balance the sustainability of structural adaptations and the dynamic maintenance of material properties.

Bone injury repair is impaired in osteoporotic individuals due to reduced osteoblast activity and increased inflammation [43,44]. In this study, Micro-CT analysis revealed that OVX rats had insufficient bone filling at the femoral drilling site and sparse regenerated bone tissue on days 10 and 21 post-injury, whereas the OVX + EX group showed significantly better bone repair, with higher bone volume fraction at the injury site. This effect presumably reflects the dual actions of exercise: it improves systemic bone metabolism by lowering bone turnover and raising E_2_ levels, thereby establishing a pro-osteogenic milieu for repair [45], and it expands the osteogenic reserve of bone tissue by mechanically priming osteoblasts and bone marrow mesenchymal stem cells to proliferate and differentiate more efficiently after injury [46]. These findings have important clinical implications: postmenopausal women are at increased risk of bone fractures, and our results suggest that pre-injury resistance exercise may improve fracture healing outcomes. This is particularly relevant for elderly individuals, who often face prolonged recovery from osteoporotic fractures.

Myostatin (MSTN) and the Wnt/β-catenin pathway are key regulators of muscle–bone crosstalk [47]. Consistent with previous reports, our study found that ovariectomy markedly increased MSTN levels in skeletal muscle and serum [18]. MSTN not only inhibits muscle growth but also binds to its receptor ActRIIB on osteoblasts and osteoclasts, activating the Smad3 pathway [25]. By activating Smad signaling, MSTN can directly repress the transcription of osteogenic genes such as Runx2 and osteocalcin and promote expression of pro-osteoclastogenic factors such as RANKL, disrupting the dynamic balance between bone formation and resorption [48,49,50]. Moreover, estrogen deficiency downregulates the Wnt/β-catenin—a canonical pro-osteogenic signal that promotes proliferation of osteoprogenitors, inhibits their apoptosis, and indirectly suppresses osteoclastogenesis by modulating the OPG/RANKL axis [51,52]. Estrogen deficiency also reduces secretion of Wnt ligands and increases expression of Wnt inhibitors such as Dickkopf-1, leading to decreased nuclear translocation of β-catenin and impaired osteoblast function [53,54]. These dual mechanisms together accelerate osteoporosis progression.

In the present study, through 10 weeks of resistance ladder-climbing training intervention followed by a 21-day detraining follow-up, our molecular detection results demonstrated that exercise can not only induce immediate molecular changes but also maintain these effects in the short term. After the intervention, rats showed reduced serum MSTN levels, suppressed quadriceps MSTN protein expression, and inhibited femoral ActRIIB/Smad3 protein expression, alongside upregulated Wnt/β-catenin protein expression. The results suggest that exercise may confer musculoskeletal protection through multiple potential targets: on one hand, it may suppress the expression of MSTN in skeletal muscle and reduce its systemic release, thereby alleviating muscle atrophy and blocking the ActRIIB-mediated Smad bone resorptive signaling pathway; on the other hand, it may inhibit the activity of GSK-3β and reduce the degradation of β-catenin, thus activating the Wnt signaling pathway, promoting the transcription of osteogenic-related genes, enhancing osteoblast function and matrix deposition, and helping to restore bone metabolic balance [55,56]. Together these effects produce a synergistic “inhibit bone resorption and promote bone formation” outcome, manifested as enhanced trabecular bone formation and improved bone biomechanical properties [57]. Notably, 21 days after exercise cessation, the expression of MSTN in the OVX + EX group remained significantly lower than that in the OVX group, while the expression of Wnt/β-catenin was still significantly elevated, which was consistent with the sustained improvement trend of bone microstructure. This suggests that the persistent regulation of these pathways may be a key mechanism underlying exercise-induced bone memory. However, future studies still need to further verify the causal roles of the MSTN and Wnt/β-catenin pathways in exercise-induced bone protection through specific pathway inhibitor experiments.

The persistence of molecular changes after exercise may be related to mechanotransduction-induced epigenetic regulation of MSTN/Wnt/β-catenin–related genes [58,59], sustaining anti-osteoporotic activity post-exercise. Osteocytes, the primary mechanosensory cells in bone tissue, perceive mechanical stimuli such as skeletal loading induced by ladder-climbing through integrins or primary cilia on their surface, and then transmit intracellular signals via downstream molecules including focal adhesion kinase and mitogen-activated protein kinases [60,61]. This signal transduction cascade may trigger epigenetic modifications such as histone acetylation in the Wnt promoter regions and DNA methylation of the MSTN gene, thereby regulating the transcription of genes related to the MSTN/Wnt pathway [60,61]. Previous studies indicate that mechanical stress–induced epigenetic modifications can maintain stable expression of regulatory genes after exercise cessation and thereby continue to influence osteoblast and osteoclast functions [40]. However, the specific epigenetic targets, the regulatory time window, and the need for maintenance exercise to consolidate these effects require further exploration.

Despite the valuable insights gained regarding the role of weight-bearing ladder climbing exercise in osteoporosis and bone injury repair in OVX rats, this study has certain limitations. First, the loading weight in this study was set as a percentage of the rats’ body weight. However, rats in the OVX group exhibited significant weight gain during the experiment, whereas those in the OVX + EX group maintained relatively stable body weight, which might have led to certain differences in the actual mechanical load borne by each group. Meanwhile, food intake was not systematically monitored throughout the intervention period, making it impossible to completely exclude the potential impact of differential food intake on body weight, muscle mass, and bone metabolism-related indicators. Future studies may adopt a fixed absolute load adjusted periodically, coupled with daily food intake monitoring and standardized feeding protocols, to further eliminate confounding factors in the experiment. Second, although post-hoc power analysis confirmed that the sample size of this study was sufficient to detect intergroup differences in the primary outcome measure (femoral BMD), the relatively limited sample size might partially restrict the accurate identification of subtle variations in secondary outcome measures with high inter-individual variability. Subsequent research could appropriately expand the sample size to improve the statistical power for secondary outcome measures, thereby verifying the effects of exercise intervention more comprehensively. Third, female SD rats were used as the animal model to simulate postmenopausal osteoporosis in this study. Given the species differences in bone remodeling patterns between rodents and humans [62], the clinical translation of the study findings requires further validation through human clinical trials. Additionally, the absence of a sham-operated + exercise group (Sham + EX group) in this study precludes a full understanding of whether the observed exercise effects are specific to estrogen deficiency or represent a general physiological adaptation applicable to all ovarian functional states. Future studies may include additional groups and conduct cross-species validation to provide a more comprehensive reference for developing clinical exercise intervention strategies. Fourth, the 21-day follow-up period in this study verified the short-term persistence of exercise effects. Extending the observation period appropriately may more clearly elucidate the long-term stability or dynamic changes in the benefits conferred by exercise, thereby providing more direct evidence for formulating long-term maintenance regimens of exercise intervention in clinical practice.

In summary, this study systematically analyzed the ameliorative effects of 10 weeks of weight-bearing ladder climbing exercise on BMD and trabecular microstructure in OVX rats, as well as the repair effect on bone injury after exercise cessation. At the phenotypic level, exercise not only effectively mitigated the decrease in serum estrogen levels, abnormal weight gain, and skeletal muscle atrophy induced by estrogen deficiency, but also significantly alleviated osteoporosis-related bone loss and improved bone strength by increasing femoral BMD, optimizing trabecular microstructure, and enhancing bone biomechanical properties; simultaneously, exercise could obviously promote the healing process of bone injury after trauma. Notably, these protective effects were partially retained 21 days after the cessation of exercise intervention. At the molecular mechanism level, exercise may provide critical signaling support for muscle growth and bone formation by sustainably inhibiting the MSTN/ActRIIB/Smad3 catabolic pathway and activating the Wnt/β-catenin anabolic pathway. It is speculated that mechanical stress may confer sustained efficacy to pathway regulation through epigenetic mechanisms such as DNA methylation and histone modification, which also explains why the ameliorative effects on musculoskeletal health persisted after exercise cessation. In addition, this study observed selective differences in exercise effects—for instance, the improvement in elastic modulus at the bone material level did not show persistence. This finding further suggests that the regulation of the musculoskeletal system by exercise is a complex, multi-targeted, and multi-dimensional process, involving not only the optimization of the systemic metabolic environment but also tissue-specific adaptive changes, thereby providing important references for future studies to further explore the precise mechanisms and optimization strategies of exercise intervention.

## 5. Conclusions

In this study, weight-bearing ladder climbing exercise may ameliorate OVX-induced bone loss by inhibiting the MSTN/ActRIIB/Smad3 pathway and activating the Wnt/β-catenin pathway. Importantly, these beneficial effects were still present 21 days after exercise cessation and enhance bone repair following injury. Our findings provide experimental evidence for the clinical application of weight-bearing ladder climbing exercise as a safe, effective, and sustainable non-pharmacological intervention for postmenopausal osteoporosis and osteoporotic bone injury.

## Figures and Tables

**Table 1:** Primer Sequences of Target Genes for Real-Time PCR.

Gene	Bidirectional Primer Sequences	Product Length	Annealing Temperature
*β-* *catenin*	R: 5′-TCA GCA CTC TGC TTG TGG TC-3′	122 bp	57.8 °C
	F: 5′-CTT ACG GCA ATC AGG AAA GC-3′		
*Smad3*	R: 5′-GCT GCA TTC CGG TTA ACA TT-3′	132 bp	58.76 °C
	F: 5′-GAG ACA TTC CAC GCT TCA CA-3′		
*ActRIIB*	R: 5′-CAT GAC ACA GCT CGT TCC AC-3′	127 bp	58.9 °C
	F: 5′-CAG AGA AAC GAG GCT CCA AC-3′		
*Wnt1*	R: 5′-GGT ACC TTT GCT GTC CTT GC-3′	226 bp	57.3 °C
	F: 5′-GTT TAC GCC ATC TCC TCA GC-3′		
*GSK3-β*	R: 5′-TTT CCA CCA ACT GAT CCA CA-3′	322 bp	55.8 °C
	F: 5′-TCT TTG GAG CCA CCG ATT A-3′		
*MSTN*	R: 5′-CCG TGG AGT GTT CAT CAC AG-3′	423 bp	51.7 °C
	F: 5′-CTG TAA CCT TCC CAG GAC CA-3′		
*GAPDH*	R: 5′-CAT TGG GGG TAG GAA CAC-3′	255 bp	55 °C
	F: 5′-CGA CTG TTA GAA CTC CCT CA-3′		

## Data Availability

The data underlying this article will be shared upon reasonable request to the corresponding author.

## Supplementary Materials

The following supporting information can be downloaded at: https://www.mdpi.com/article/10.3390/biology15010055/s1, Table S1: The original Western blot images for Figure 5; Table S2: The original Western blot images for Figure 9.

## Abbreviations

The following abbreviations are used in this manuscript:

OVX	ovariectomized
MSTN	myostatin
BMD	Bone Mineral Density
BV/TV	Trabecular Bone Volume fraction
ALP	Alkaline Phosphatase
TRACP	Tartrate-Resistant Acid Phosphatase
Tb.Th	trabecular thickness
Tb.Sp	trabecular separation
Micro-CT	micro-computed tomography
E_2_	estradiol
Tb.N	trabecular number
