# Supplementary material for: Weight-Bearing Ladder Climbing Exercise Improves Bone Loss and Bone Microstructural Damage While Promoting Bone Injury Healing in OVX Rats
Source: Biology (Basel). 2025 Dec 28;15(1):55. doi: 10.3390/biology15010055 (PMC12785010; doi:10.3390/biology15010055)
Supplement: Supplementary file 1 [file biology-15-00055-s001.zip › biology-4037053-supplementary.pdf]

Supplementary figures of western blot

Table S1: The original western blot images for Figure 5.

Housekeeping proteins	Objective proteins	Build-Up
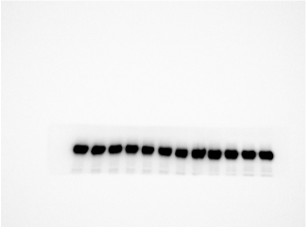	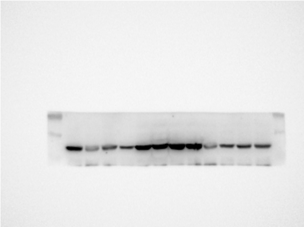	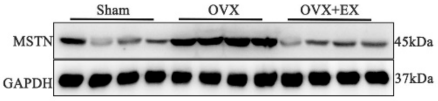
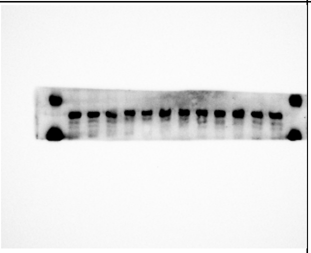	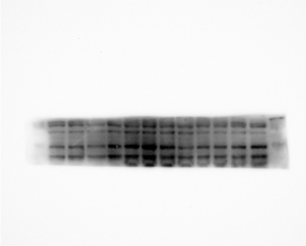	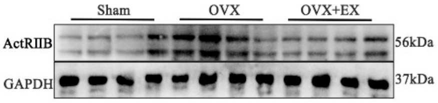
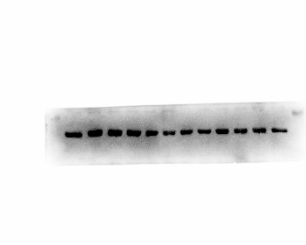	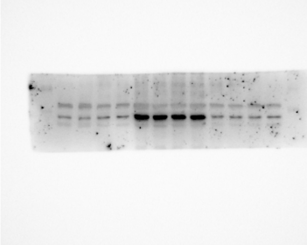	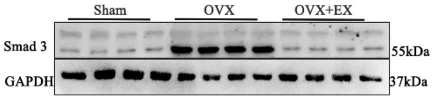
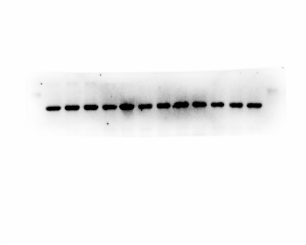	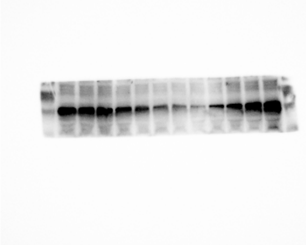	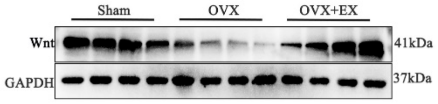
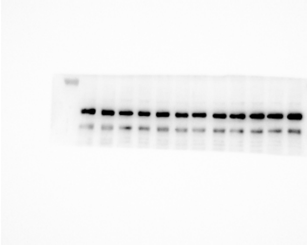	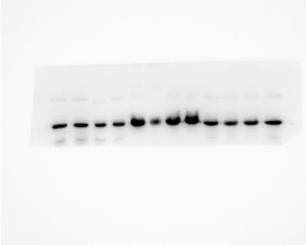	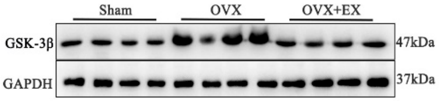
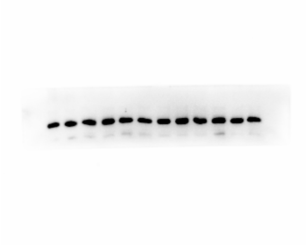	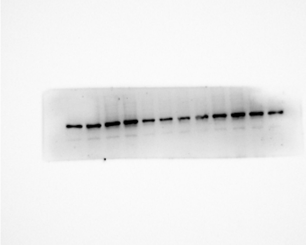	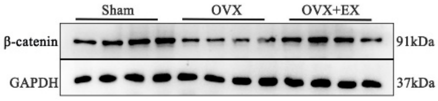

Table S2: The original western blot images for Figure 9.

Housekeeping proteins	Objective proteins	Bilud-Up
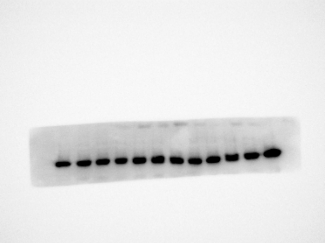	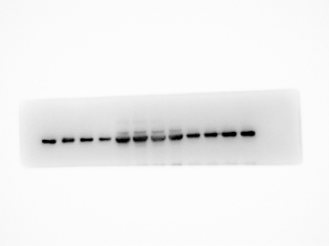	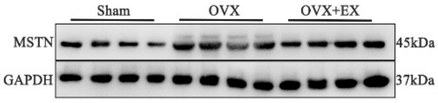
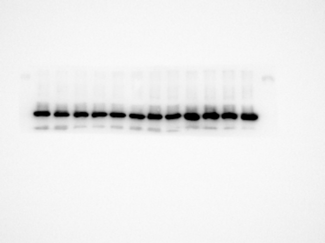	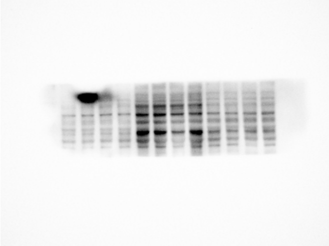	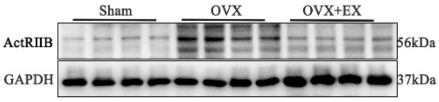
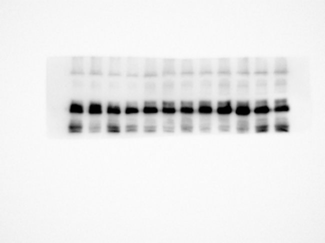	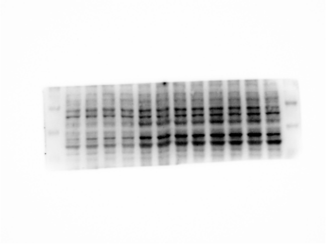	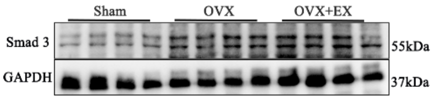
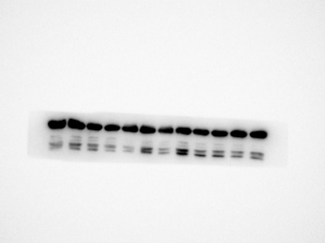	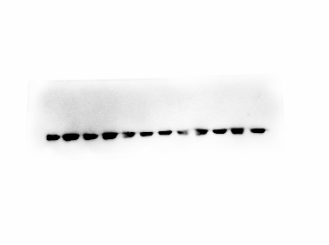	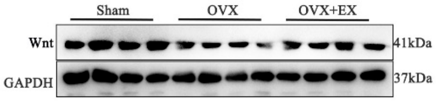
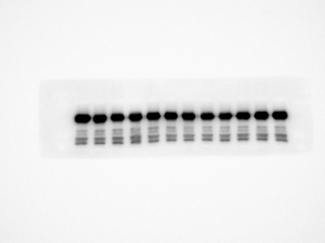	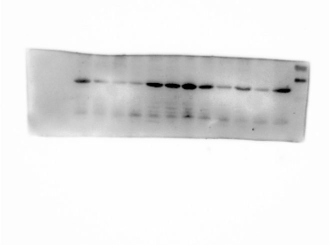	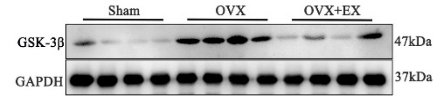
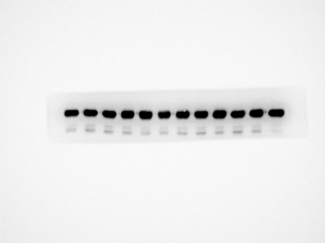	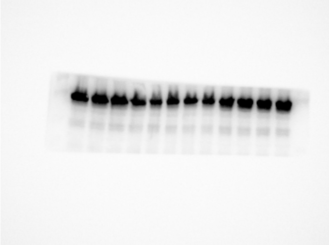	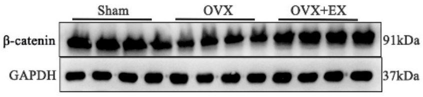
